# Long-Term Survival, Prognostic Factors, and Quality of Life of Patients Undergoing Pelvic Exenteration for Cervical Cancer

**DOI:** 10.3390/cancers14092346

**Published:** 2022-05-09

**Authors:** Mihai Stanca, Dan Mihai Căpîlna, Mihai Emil Căpîlna

**Affiliations:** First Obstetrics and Gynecology Clinic, University of Medicine, Pharmacy, Science and Technology “G.E. Palade” of Târgu Mureș, Gheorghe Marinescu Street, Number 38, 540142 Târgu Mureș, Romania; capilnadan@gmail.com (D.M.C.); mcapilna@gmail.com (M.E.C.)

**Keywords:** cervical cancer, survival, pelvic exenteration, quality of life, QLQ-C30, QLQ-CX24

## Abstract

**Simple Summary:**

Cervical cancer is a malignancy that can easily be prevented through the simplest means of investigation. However, every two minutes a woman dies worldwide due to cervical cancer. Upon closer inspection, the reasons for death include low compliance with screening programs and HPV vaccination, inadequate information, media coverage, ethnicity, conviction, and misinformation. In Europe, Romania has a forefront position regarding the incidence of, and mortality caused, by this disease. Despite the many efforts carried out to counteract this situation, cervical cancer has become an ongoing health issue that still needs debating, particularly in the developing countries of Eastern Europe. Consequently, and for the first time in Romania, the authors of this study aim at revealing, through scientifically validated elements, the prognostic factors that influence the survival of patients undergoing pelvic exenteration for FIGO stage IVA, recurrent or persistent cervical cancer after previous conclusive treatment. The study also rigorously assesses the quality of life, using validated questionnaires, placing Romania alongside those countries that have highlighted these issues and sought to find answers.

**Abstract:**

Background: Considerable efforts have been carried out over the past 30 years to support patients with advanced cervical cancer. Throughout this time, Eastern European countries have been left aside from the decision-making groups on this matter, hence the absence of similar studies in this geographical area. In these countries, the quality of life (QoL) of patients with cervical cancer might be considered a “caprice”, and the discomforts they encounter following pelvic exenteration for cervical cancer are often perceived as a “normal phenomenon”. Methods: This study examined forty-seven patients submitted to pelvic exenteration followed up for nine years after the surgical intervention. The first objective of this study is to identify the prognostic factors that influence the overall survival (OS) of patients undergoing pelvic exenteration for FIGO stage IVA, recurrent or persistent cervical cancer after previous conclusive treatments. The second objective is to assess the QoL of the surviving patients using the QLQ-C30 and QLQ-CX24 standardized questionnaires. Results: The mean age of the participants was 54 years (range 36–67). At the time of the study, there were 25 living patients (53.2%), the 3-year OS was 61%, and the 5-year OS was 48.7%. Cox regression analysis recognized parameter invasion, pelvic lymph node metastases, positive resection margins, early postoperative complications, and infralevatorian pelvic exenteration as negative prognostic factors influencing the OS (*p* < 0.05). Of the 25 survivors, 18 patients answered the QoL questionnaires. The cost of favorable survival has been translated into poor overall QoL, unsatisfactory functional, social, and symptom scores, a high prevalence of cervical cancer-specific symptoms such as lymphedema, peripheral neuropathy, severe menopausal symptoms, distorted body image, and lack of sexual desire. The lower scores are comparable to the only three studies available in the literature that assessed the QoL of patients undergoing pelvic exenteration precisely for cervical cancer. Conclusions: Despite its retrospective nature and some limitations, this paper, similar to other studies, shows a decent OS but with a marked adverse impact on QoL, suggesting the importance of adequate psycho-emotional and financial support for these patients following pelvic exenteration. This study also contributes to the current knowledge regarding advanced cervical cancer treatment, depicting survival, prognostic factors, and QoL of patients undergoing pelvic exenteration for cervical cancer in a reference center in Eastern Europe. Our study can provide a comparison for future prospective randomized trials needed to confirm these results.

## 1. Introduction

Brunschwig first described pelvic exenteration in 1948 as a palliative procedure for patients with advanced pelvic malignancies [1]. This procedure involves the removal of the reproductive organs, the rectum or bladder or both, and lymph nodes in the pelvis. Since then, pelvic exenteration has become a routine surgical procedure—an option of choice with long-term benefits for treating pelvic recurrences [2].

Today, most indications for pelvic exenteration are recurrent or persistent cervical cancer cases after radiochemotherapy [3,4,5,6,7]. There are few treatment options available when local recurrence arises due to pelvic irradiation for cervical cancer. Consequently, the re-irradiation of the same anatomical site is prohibited. At the same time, chemotherapy by itself is ineffective in controlling recurrences confined in the tissue previously exposed to radiotherapy, which usually tends to be less vascularized [8,9]. Regarding advanced primary tumors, the superiority of pelvic exenteration over radiotherapy or radiochemotherapy has not yet been confirmed [9].

Despite improving surgical techniques and postoperative management, such a complex surgical procedure is still linked to a high rate of complications and an unfortunate QoL. Though, when R0 resection can be attained, there are acknowledged survival benefits [10]. R0 surgical resection is achievable in more than 70% of cases and remains one of the most critical determinants for survival [11,12]. For this reason, pelvic exenteration is becoming a beneficial choice for a growing number of recurrent cervical cancer occurrences.

Considerable efforts have been made over the past 30 years to support patients with advanced cervical cancer. Large-scale randomized studies have been conducted in the United States, China, Japan, and especially in southern, western, and northern Europe to identify the negative prognostic factors and the QoL of these patients and the most effective therapeutic methods. However, European “Eastern Bloc” countries have been left out of decision-making committees regarding these issues, resulting in the absence of similar studies in this geographical area. In these countries, the QoL of patients with cervical cancer might be considered a “caprice”, and the difficulties patients encounter following pelvic exenteration for cervical cancer are seen as a “normal phenomenon”. Therefore, this study aims at two objectives. The first objective was to identify the prognostic factors that influenced the overall survival (OS) of patients undergoing pelvic exenteration for the International Federation of Gynecology and Obstetrics (FIGO) stage IVA, recurrent or persistent cervical cancer after previous conclusive treatments. The second objective was to assess the QoL of the surviving patients using two standardized questionnaires issued by the European Organization for Cancer Research and Treatment (EORTC), namely the Quality of Life Questionnaires - QLQ-C30 [13] and QLQ-CX24 [14].

The need for this research lies in the absence of comparative studies conducted in Eastern European countries, where the survival and QoL of these patients are unknown.

## 2. Materials and Methods

This study was conducted with the agreement of the Ethics Committee of our Institute (approval code: 34535/13.12.2019). In addition, an EORTC agreement was obtained to use the QoL Questionnaires QLQ-C30 and QLQ-CX24. Written informed consent of patients included in the study was also obtained.

This observational retrospective study included 47 patients who met the inclusion criteria. The pelvic exenterations were performed for FIGO stage IVA, recurrent or persistent cervical cancer after definitive concomitant radiochemotherapy ± radical hysterectomy (Table 1 and Table 2).

After the surgical treatment, the patients presented for regular follow-up visits quarterly for the first two years, biannually for the next three years, and then annually until November 2020. Before each medical visit, patients fulfilled complete laboratory examinations and an annual thoracoabdominal CT or MRI examination. During the medical visits, local and general clinical examinations, pap-smear of the vaginal vault, and ultrasound examinations were performed.

The deaths were recorded by telephone or postal cards obtained from the patient’s relatives.

### 2.1. Inclusion and Exclusion Criteria

The inclusion criteria consisted of the following: patients with histologically, clinically, and proven imagistic FIGO stage IVA (bladder and/or rectal invasion), recurrent or persistent cervical cancer subjected to pelvic exenteration, with a minimum follow-up period of 24 months.

The QoL study did not include patients who died or had disease recurrences or other known malignancies, nor those who refused participation or whose follow-up was lost.

Also, to confirm pelvic exenteration’s feasibility and exclude the oncological contraindications, all the patients underwent exhaustive preoperative imaging investigations (transvaginal ± transrectal ultrasound, thoracoabdominal CT, and pelvic MRI, and when needed, colonoscopy and cystoscopy). Patients with paraaortic lymphatic metastases were excluded from the study to avoid aberrant survival and QoL outcomes.

### 2.2. Treatment Administration

The patients with recurrent and persistent disease included in the study received prior radical hysterectomy and adjuvant radiotherapy or concomitant definitive radiochemotherapy. In contrast, the patients with FIGO stage IVA cervical cancer, most with vesicovaginal or rectovaginal fistulas, had no prior treatment. All the patients received a pelvic exenteration, completed with adjuvant chemotherapy if positive margins were found on the specimen. Chemotherapy regimens consisted of weekly administration of Cisplatin ± Fluorouracil.

All procedures were performed by a highly experienced team in ultra-radical procedures with curative, not palliative intention [15].

Anterior pelvic exenteration consisted of the removal of the bladder, urethra, partial or total resection of the vagina, removal of the uterus or vaginal vault; posterior pelvic exenteration involved removal of the rectum with or without anal resection, partial or total resection of the vagina, removal of the uterus or vaginal vault; and total pelvic exenteration required removal of the bladder, partial or complete resection of the vagina, removal of the uterus or vaginal vault, removal of the rectum with or without anal amputation. An omental flap was used to cover the pelvis. Pelvic lymphadenectomy was performed in all cases where the lymph nodes were not removed during a radical hysterectomy.

Proper preoperative measures were carried out. Patients with postoperative hemoglobin levels of ≤8 g/dL received blood transfusions. In addition, histopathological examinations were performed for each surgical sample collected.

### 2.3. Statistical Analysis and Data Assessment

The categorical variables were analyzed using the Chi-square test, and the differences between the variables were analyzed using the *t*-test; *p* < 0.05 was considered statistically significant. OS was assessed according to the Kaplan–Meier method. Survival was defined as the period elapsed from the time of surgery to the patient’s last contact. The date of death was the final objective of the survival analysis. Survival was compared using the Cox regression and the log-rank test, thus identifying the risk factors that influenced the survival.

The proportional hazard examination model was used to identify and evaluate the prognostic factors on the 5-year survival, with a 95% confidence interval.

The software used for the statistical investigation was IBM SPSS 23.0.California.

### 2.4. Quality of Life Questionnaires

All living patients who met the inclusion criteria received a postal letter containing an informed consent to participate in the study and a set of translated QoL questionnaires issued by the European Organization for Research and Treatment of Cancer (EORTC)—the “Quality of Life Questionnaire—QLQ-C30” and “Quality of Life Questionnaire—QLQ-CX24”, together with a postage-paid envelope ready for the return of the completed questionnaires. Patients who did not respond within three months received another letter containing the same items mentioned above and a call a month later.

The EORTC QLQ-C30 questionnaire [13] is generally used for all malignancies. It consists of 30 elements: five functional scales (role, physical, cognitive, social, and emotional), three symptom scales (pain, nausea/vomiting, and fatigue), a scale for measuring the overall QoL, and six individual items evaluating the symptoms often reported by patients with malignancies (loss of appetite, insomnia, dyspnea, diarrhea, and constipation), and the self-reported financial difficulty.

The EORTC QLQ-CX24 questionnaire [14] accompanies the previous one and is specific for cervical cancer. It includes 24 items related to symptoms and possible outcomes following cervical cancer treatment. It has four functional scales consisting of two multi-item scales (sexual/vaginal functionality and body image); two single-item scales (sexual pleasure and sexual activity); five symptom scales; four single-item scales (sexual anxiety, peripheral neuropathy, menopausal symptoms, and lymphedema); and one multi-item scale (symptom experience) [14]. To independently assess functionality and symptoms, both questionnaires were answered on a 4-point scale as follows: “Not at all”, “Little”, “Quite little” and “Very much”, as well as a 7-point scale, from poor to excellent that assesses health and QoL during the last week [13,14].

To facilitate questionnaire interpretation, the responses were converted on a scale from 0 to 100 using their interpretation manual [13]. For the EORTC QLQ-C30 questionnaire, a higher score for the overall QoL and functional scales suggests satisfactory functionality and a superior QoL. For symptom items and scales, a higher score results in symptoms of high intensity [13]. For the EORTC QLQ-CX24 questionnaire, high scores show severe symptoms and poor functionality, except for sexual activity and satisfaction, when a higher score indicates better results [14]. All results were depicted in raw numbers and rates, summarized as medians [ranges] and mean (SD) (95% standard error).

## 3. Results

The authors identified 47 patients who met the inclusion criteria for this study. All patients underwent pelvic exenteration for cervical cancer at the Emergency County Hospital of Târgu Mures, Romania, Department of Obstetrics-Gynecology I, between January 2010 and March 2019.

**Figure 1:**
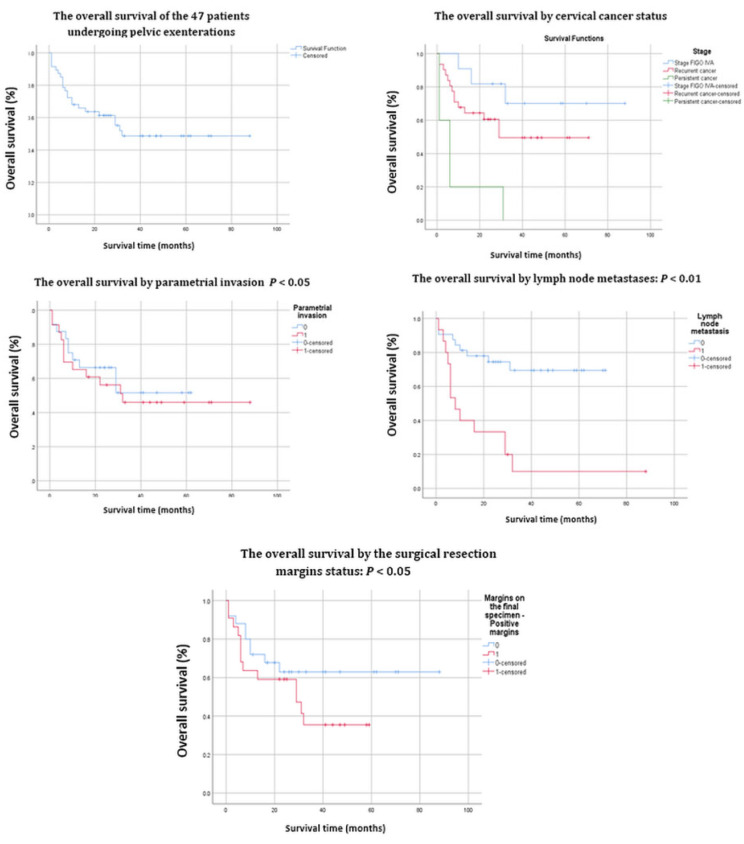
Kaplan–Meier survival curves of the 47 patients who had undergone pelvic exenterations.

The average participant’s age was 54 years (36–67). 23.4% of patients had FIGO stage IVA, 66% had recurrent cancer, and 10.6% had persistent cervical cancer after previous conclusive treatment. Squamous cell carcinoma was the most common histological type with 85.1% of cases, followed by adenocarcinoma with 14.9%. Cox regression analysis recognized parametrial invasion, pelvic lymph node metastases, positive resection margins, early postoperative complications, and infralevatorian pelvic exenteration as negative prognostic factors influencing the OS (*p* < 0.05). (Figure 1). Accordingly, 23 patients (48.9%) had parametrial invasion, 15 patients (31.9%) had pelvic lymph node metastases, and 22 patients (46.9%) had positive resection margins.

Regarding treatment regimens, 11 patients (24%) did not receive any prior therapy before pelvic exenteration, 2 patients (4%) underwent radical hysterectomy before pelvic exenteration, 34 patients (72%) received concomitant definitive radiochemotherapy prior to pelvic exenteration, and 13 patients (23%) underwent adjuvant chemotherapy following pelvic exenteration (Table 2).

Concerning the type of exenteration, topographically, 29 patients (61.7%) underwent total pelvic exenteration, 17 (36.2%) anterior pelvic exenteration, and only one patient (2.1%) received posterior pelvic exenteration. There were 28 (59.6%) supralevatorian exenterations and 19 (40.4%) infralevatorian exenterations regarding the levator ani muscle (Table 2).

Patients undergoing total and anterior pelvic exenteration required reconstruction of the urinary tract by performing a Bricker-type incontinent ileal or sigmoid derivation (46 patients—98%). When the reconstruction of the intestinal tract was required, colostomy was performed in 27 cases (90%) and colorectal anastomosis in 3 patients (10%).

No significant intraoperative complications were reported with particular attention to anatomical vascular variations [16]. However, the authors account for postoperative complications in 27 patients (57%). Accordingly, there were 18 (38.3%) early complications (Table 2). Namely, three deaths occurred in the first month following the procedure (two patients developed acute pulmonary edema, and one patient developed stercoral peritonitis by cecal perforation with severe sepsis). The rest of the early complications were managed by re-laparotomies with favorable evolution, being discharged after a more extended period of hospitalization (Table 2). Nine (19.1%) late complications (Table 2) were reported, which were also managed by re-laparotomies with satisfactory results.

The average operative time was 360 min, the approximate amount of blood loss was 1.100 mL, and the mean transfused volume was 675 mL. The average period of hospitalization following pelvic exenteration was 19 days.

The follow-up time of patients after the radical intervention was 44.5 months (1–88 months). At the time of the study, there were 25 living patients (53.2%), the 3-year OS was 61%, and the 5-year OS was 48.7%. All information is detailed in Table 2.

### The Results of the Quality of Life Study

Of the 25 living patients, 72% (*n* = 18) answered the QoL self-assessment questionnaires. Failure to contact patients was caused by address or phone number changes.

Therefore, the QoL study population included 18 patients with a mean age of 53 years (range 36–66 years). Out of these, 14 patients (78%) received conclusive concomitant chemotherapy prior to pelvic exenteration, and 4 patients (22%) received adjuvant chemotherapy following the surgical procedure. The questionnaires were sent to patients after an average follow-up time of 26 months post-treatment.

Regarding the EORTC QLQ-C30 questionnaire [13], a primary tool universally used for all cancers, the survivors showed an unsatisfactory overall QoL with an average score of 32.6 out of 100. In addition, the functional status characterized by physical, role, cognitive, emotional, and social scales also had unsatisfactory scores of 53.4, 28.5, 46.4, 37.0, and 51.8, respectively, indicating poor functionality and QoL.

**Figure 2:**
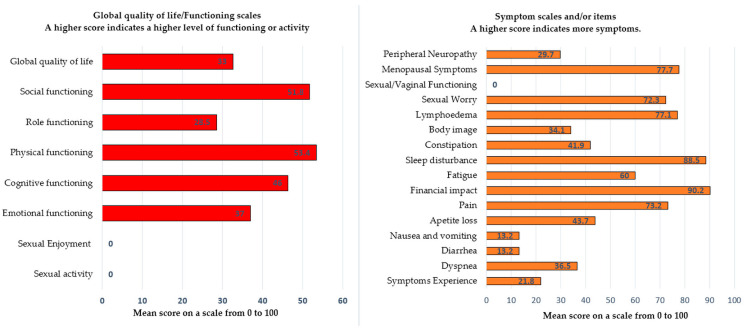
The QoL of the 18 patients who answered the EORTC QLQ-C30 and EORTC QLQ-CX24 who underwent pelvic exenteration.

The symptom scales consisting of nausea/vomiting, dyspnea, pain, diarrhea, loss of appetite, and financial difficulties are associated with an increased level of side effects related to cancer treatment (Table 3) (Figure 2). 

The symptoms that triggered the most common distress were constipation, insomnia, fatigue, and loss of appetite, with scores of 41.9, 88.5, 60.0, and 43.7, correspondingly (Table 3) (Figure 2). Also, the advanced stage of the disease and its treatment led to financial impairments with a high score of 90.2.

The EORTC QLQ-CX24 questionnaire [14] assesses the explicit symptoms of cervical cancer. The results of this highlight the unfavorable influence of pelvic exenteration on the QoL with a score of 21.8. In addition, body image, lymphedema, peripheral neuropathy, and menopausal symptoms obtained scores of 34.1, 77.1, 29.7, and 77.7, respectively, indicating a high level of cervical cancer-specific symptoms following the oncological therapy (Table 3) (Figure 2).

Regarding sexual activity, the data reveal an increased level of sexual concern, with a score of 72.3 ± 28.8. However, no patient confirmed sexual activity following pelvic exenteration. Thus, questions regarding sexual activity, sexual/vaginal function, and sexual satisfaction were left unanswered. Out of the 18 patients who responded to the QoL questionnaires, 11 (61%) underwent supralevatorian pelvic exenteration, and 7 patients (39%) underwent sublevatorian pelvic exenteration, which involved total resection of the vagina, with unfeasibility of traditional sexual intercourse.

## 4. Discussion

### 4.1. Survival and Prognostic Factors

Over the last decade, the number of pelvic exenterations has increased significantly. However, these surgical procedures are linked to high rates of perioperative morbidity (>50%) [9,10,18].

Several acknowledged risk factors influence survival, including tumor size [3,8], the period without recurrences after primary treatment [8], histological subtypes [19], and lymph node metastases [4,20], as well as lymphovascular invasion [21]. In the current study, survival was influenced by parameter invasion (*p* 0.059) (micrometastases in the fibrous structures connecting the uterus to the pelvic walls), positive resection margins (*p* 0.052), and lymph node metastases (*p* 0.017), similar to patients undergoing radical hysterectomy for cervical cancer [22,23].

Topographically, in this study, 29 patients (61.7%) underwent total pelvic exenteration, 17 patients (36.2%) underwent anterior pelvic exenteration, and only one patient (2.1%) undertook posterior pelvic exenteration with a 5-year OS of 46.7%, 50.3 %, and 100% accordingly.

Magrina et al. [24] proposed the subclassification of pelvic exenterations by the levator ani muscle, which has been helpful for improved, broader understanding of pelvic resection. Thus, type I (supralevatorian), type II (infralevatorian), and type III (infralevatorian with vulvectomy). Of the patients studied, 28 (59.6%) underwent supralevatorian pelvic exenteration with a 5-year OS of 59.6%, and 19 patients (40.4%) underwent infralevatorian pelvic exenteration with a 5-year OS of 43.9% (*p* < 0.02). Patients in the latter group had a poorer OS and QoL, with ten of these patients experiencing early postoperative complications, which in two cases led to death in the first month. An explanation might be that tumors involving the inferior anatomical pelvic structures (distal vagina and urethra, vulva, levator ani, or obturator muscles) usually need a more contentious procedure with more extensive removal of pelvic structures, consequently leading to more postoperative complications. Thus, it is not the infralevatorian pelvic exenteration procedure per se that leads to higher death rates but the complications accompanying it.

A permanent colostomy is usually performed to restore bowel function. If preservation of the anal sphincter is preferred, a colorectal/coloanal anastomosis may be considered to restore continuity. Lago et al. [25] performed a permanent colostomy on 78% of patients in their study. The authors of the current research performed permanent colostomy in 27 patients (90%) and colorectal anastomosis in only three cases (10%).

For urinary tract restoration, the techniques can be classified into two groups: incontinent diversion (ileal or sigmoid reservoirs) or continent diversion (heterotopic reservoirs and orthotopic neobladder connected to the urethra-like Budapest pouch [26]). Some authors have determined no differences in postoperative complications between the two groups [27,28]. All 46 patients in the current study who required urinary tract restoration received incontinent Bricker ileal or sigmoid diversion. Only one case did not require urinary tract repair as the patient underwent posterior pelvic exenteration.

Pelvic exenterations are burdened with high rates of postoperative complications [9,10,18]. Thus, grade 3, 4, and 5 complications according to the Clavien–Dindo scale [29] are observed in approximately 60% of cases [18], with 10% of patients requiring re-laparotomies [30]. Matsuo and Lewandowska [2,31] identified several common postoperative complications—hemorrhage (31.8% of cases); ileus (25.8%); complications of the surgical wound (21.3%); and respiratory failure (16.1%) as well as sepsis, thromboembolism, heart failure, shock, fistulas, and abscess. In a study by Jalloul et al., the most commonly observed complications were wound dehiscence (approximately 55% of cases), urostomy complications, and abscess [18].

Yoo et al. [20] reported a total morbidity rate of 44%; 10 patients (16%) had early complications (30 days or less after pelvic exenteration), while 22 patients (36%) had late complications. Difficulties with surgical wounds represented the most common early complications (7/18), and intestinal fistulas were frequent late complications (9/30).

Similar to the mentioned studies, a similar profile of early postoperative complications was observed in the present study. Thus, 18 patients (38.3%) suffered early postoperative complications (pelvic abscess; entero-perineal, entero-vaginal, and entero-ileal fistulas; wound infections; intestinal occlusions; acute pulmonary edema; peritonitis; sepsis; acute renal failure; urine loss through Bricker ileal bypass; and pneumonia). The two patients with acute pulmonary edema and the patient with sepsis died in the first postoperative month. The rest of the complications were managed by re-laparotomy with favorable results. It should be observed that patients who suffered early complications had an unfavorable prognosis (*p* 0.007).

The late complications rate was lower (*n* = 9) (19.1%), including eventrations; bilateral ureteral-ileal stenosis; entero-perineal fistulas; small bowel syndrome; colostomy stenosis; and Bricker urinary tract fistula. Also, all cases were surgically managed by re-laparotomies.

The average perioperative mortality rate (within 30 days after the procedure) is about 2% [32]. Jalloul et al. [18] reported 60–95% perioperative morbidity rates and mortality rates up to 5%. By contrast, Matsuo et al. saw a reduction in the mortality rate over the last decade from 4.0–7.2% to 1.9–2.3% of cases [31]. The perioperative mortality rate experienced in the current study group was 6% (*n* = 3).

The long-term results of pelvic exenteration patients depend on the final surgical outcome. The goal is obtaining R0 resection (free surgical margins). When R0 is achieved, the 3-year OS survival rate is about 50% [32]. Thus, R0 resection remains the most crucial determinant of survival [11,32,33]. De Gregorio et al. observed a 5-year OS of 34.4% when R0 was reached [10], while Li et al. found that a positive resection margin negatively and independently impacts the OS [12]. Accordingly, R1 resection has been associated with a significantly worse prognosis, with a mean OS of 10.4 months [34]. Modern imaging does not accurately identify the local microscopic disease and is inadequate for preoperative resection planning [35]. Patients in this study with negative resection margins (64%, *n* = 30) had a 5-year OS of 62.9% compared to those with R1 (36%, *n* = 17) who had an OS of 33.5% (*p* 0.052). Positive resection margins were most commonly found in the rectum, vagina, and urethra. The current tendency of the authors is to extend the excision as low as necessary to the infralevator ani level, straining at the same time to avoid postoperative complications which, as mentioned above, are common in this group of patients. Hence, pelvic exenteration may provide locoregional control in patients with no abdominal tumoral spread and where R0 resection may be achieved.

Although pelvic exenteration is a complex surgical procedure that requires a prolonged surgical period and is often linked to a high risk of complications, the benefit of survival justifies its use in daily practice [36].

The 5-year survival ranges from 30 to 60% [3,5,27,35,37,38] despite a high morbidity rate. In 2019 Lago et al. [25] reported a 4-year OS of 41.6% over an average follow-up time of 18.5 months. Yoo et al. found a 5-year OS of 56% [20]. In 2020, Lewandowska et al. [2] observed a slightly lower long-term survival rate. However, in a selected group of patients, such as the squamous cell carcinoma group, 1/3 of the patients survived for more than three years, and ¼ survived for more than 4.5 years [2]. The 3-year OS of the patients in this study was 61%, while the 5-year OS was 48.7%.

It is essential to make a thorough preoperative evaluation, highlighting the consequences of this radical surgery [25].

### 4.2. Quality of Life

Although according to the latest guidelines issued by the European Society of Gynecological Oncology in 2018 [39], pelvic exenteration remains the only curative option for patients with persistent or recurrent cervical cancer, the impairment of the patient’s QoL and the high-risk surgical complications continue to be a significant concern. Therefore, one of the essential decisional factors that qualify the patient for pelvic exenteration is the correlation between cervical cancer recurrence and the appearance of clinical symptoms. These signs include chronic pain and vaginal fistulas. The latter has been shown to negatively impact the patient’s QoL [32].

The QoL of patients following pelvic exenteration has been investigated in a scarcity of studies [40,41,42]. Except for Magrina Dessole et al. [40], the other two studies did not use the validated questionnaires EORTC QoL C-30 and CX-24. Like our study, none of them had a control group, making it challenging to identify the cause and effect of an unfavorable QoL, as it may be influenced by the cervical cancer diagnosis per se, the pelvic exenteration, the recurrences or persistence of the disease, or by the history of radiochemotherapy. Among the independent predictors of a poor QoL are a permanent colostomy and an incontinent bladder, which lead to financial difficulties, a weak attitude towards the disease, gastrointestinal symptoms, insomnia, etc. [40]. Hawighorst-Knapstein et al. [42] proved a significantly lower QoL in women with two ostomies compared to those without. These findings strongly emphasize the need for more efforts to achieve a complete reconstruction of the gastrointestinal tract to ensure higher QoL standards [40].

The overall QoL of the patients in this study was deeply affected, with a value of 32.6. The functionality represented by the role, physical, cognitive, emotional, and social scales attained scores of 28.5, 46.4, 37.0, 51.8, and 28.5, respectively. These scores can range from 0 to 100, with a higher score signifying a higher grade of functionality. These results are more unfavorable than those obtained by Magrina Dessole et. al, who reported an overall QoL of 64.6 [40]. The latter study was conducted in Rome, where patients undergoing radical treatments for malignancies can benefit from advanced psycho-emotional counseling and support programs included in their medical insurance.

Consequently, this QoL discrepancy may also occur because the patients with malignancies in our country tend to have depressive disorders linked to the cancer diagnosis, which may be further worsened after the pelvic exenteration in the absence of proper support offered by the family and the healthcare programs. However, Youssef et al. [41] suggest that although the QoL declines immediately after pelvic exenteration, most patients adjust well both physically and psychologically, restoring most areas that define the QoL after a period of 12 months. Half of the patients reported feeling comfortable 12 months after pelvic exenteration, compared to only 19% at the 3-month interview [41]. When counseling patients, it is essential to emphasize that the “baseline quality of life” refers to the QoL before the patient undergoes pelvic exenteration [43].

In terms of symptom scales, most of the current study patients experienced insomnia, fatigue, and pain with similar scores compared with the other three studies [40,41,42]. However, the financial impact caused by the cancer treatment was more remarkable, with a score of 90.2. Of the patients, 61.7% originate from rural areas with limited financial resources. Also, the economic impact may have influenced some aspects of the functional and symptom scales.

Focusing on the sexual life aspects, it is essential to note that the average age of the 18 patients who answered the QoL questionnaires in our study was 53 years (36–66 years), with 67% (*n* = 12) originating from rural environments. Of the 18 patients, 11 (61%) underwent supralevatorian pelvic exenteration, and 7 patients (39%) underwent sublevatorian pelvic exenteration, which assumed total resection of the vagina, an unfeasible condition for regular sexual intercourse. However, none of the 18 patients answered any questions regarding their sexual life. It is conceivable that the avoidance of answering these questions is deliberate, as these issues are a taboo subject for many of our country’s patients, both for ethnic and religious explanations.

Magrina Dessole et al. [40] state that roughly 25% of the women included in their study who were submitted to pelvic exenteration without vagina removal were sexually active with reasonable pleasure levels. Given the complexity of sexual problems, it seems reasonable to perform a supralevatorian pelvic exenteration while maintaining the vagina [44]. In carefully selected cases, vaginoplasty techniques may be provided to support decent sexual activity [45,46].

In addition, our patients’ impaired QoL ratings suggest the importance of adequate psycho-emotional and financial support for these patients following pelvic exenteration.

### 4.3. Strengths and Limitations of the Study

One of the strengths of this research is its uniqueness in Eastern European countries.

The study includes a relatively high number of patients (*n* = 47) who were followed for an average of 44.5 months (1–88), targeting two main objectives. The first objective was to identify elements that influenced the 5-year OS and perform a detailed analysis of all demographic, clinical, surgical, and histopathological data and oncological treatments received. The second objective was to assess the QoL of 18 of the 25 living patients who answered the EORTC QLQ-C30 and EORTC QLX-CX24 validated questionnaires. Despite the group’s heterogeneity, the analysis describes a poorer QoL compared with other similar studies [40,41,42].

The limitations of the study lie in its retrospective observational nature. In addition, there is heterogeneity in the treatment received by patients. The study did not have a control group (e.g., healthy participants) or a pre-treatment assessment of the QoL. The analysis was not performed separately according to the different treatment regimens received.

## 5. Conclusions

Although, according to the latest guidelines issued by the European Society of Gynecological Oncology in 2018 [39], pelvic exenteration remains the only curative option for patients with persistent or recurrent cervical cancer, the impairment of the patient’s QoL and the high-risk surgical complications continue to be a significant concern. However, the benefit of survival justifies using this procedure in daily practice [36].

This study, unique in Eastern European countries, demonstrates a 3-year OS of 61% and a 5-year OS of 48.7%, similar to other published studies which declared 5-year survival ranging between 30% to 60%. However, the cost of favorable survival has been translated into poor overall QoL, unsatisfactory functional, social, and symptom scores, and a high prevalence of cervical cancer-specific symptoms such as lymphedema, peripheral neuropathy, severe menopausal symptoms, distorted body image, and lack of sexual activity. These results suggest the importance of adequate psycho-emotional and financial support for these patients following pelvic exenteration.

Regardless of its limitations, similar to other studies, this research makes a reliable contribution to the current knowledge regarding advanced cervical cancer, providing a depiction of survival, prognostic factors, and QoL of patients undergoing pelvic exenteration for cervical cancer in a reference center in Eastern Europe. In addition, this article provides a basis for future prospective randomized trials needed to confirm the results.

## Figures and Tables

**Table 1:** The characteristics of the 47 patients who underwent pelvic exenterations for cervical cancer, their prognostic factors and 5-year OS.

	Number (%) or Median (Range)	Overall Survival			
		5-Year Survival Rate	95% CI	Mean Survival (Months)	*p-*Value
**No. of Patients**	47				
**Age (Years)**	54 (36–67)				
30–40	2 (4.3%)	50.0%	53.5–46.4	10.5	0.126
41–50	14 (29.8%)	61.2%	62.6–59.8	59.8	0.062
51–60	19 (40.4%)	36.2%	37.3–34.9	35.2	0.294
61–70	12 (25.5%)	58.3%	59.7–56.8	37.8	0.584
**Provenance**					0.940
Urban	18 (38.3%)	50.0%	51.1–48.8	39.0	
Rural	29 (61.7%)	49.6%	50.6–48.5	51.6	
**Cancer status/Figo stage**					
IVA	11 (23.4%)	70.1%	71.5–68.9	67.8	0.220
Recurrent cancer	31 (66.0%)	49.6%	50.6–48.5	41.3	0.167
Persistent cancer	5 (10.6%)	0%		9.0	0.665
**Tumor size**					
<4 cm	24 (51.1%)	58.4%	59.5–57.2	46.6	0.460
≥4 cm	23 (48.9%)	38.3%	39.4–37.1	41.8	0.468
**Histology**					
Squamous Cell Carcinoma	40 (85.1%)	49.2%	50.1–48.3	49.7	0.356
Adenocarcinoma	7 (14.9%)	42.9%	44.7–41.0	35.2	0.356
**Tumor Differentiation Grade**					
Grade 1 (Well Differentiated)	8 (17.0%)	21.9%	23.8–19.9	34.3	0.795
Grade 2 (Moderately Differentiated)	14 (29.8%)	56.3%	57.6–54.9	43.8	0.996
Grade 3 (Poorly Differentiated)	25 (53.2%)	50.8%	51.8–49.7	48.6	0.848
**Depth of Cervical Stromal Invasion**					
Inner 1/3	4 (8.5%)	50.0%	52.5–47.5	23.7	0.664
Middle 1/3	14 (29.8%)	46.8%	48.3–45.2	38.5	0.222
Outer 1/3	29 (61.7%)	48.4%	49.4–47.4	48.0	0.170
**Lymphovascular Space Invasion**					
Positive	26 (55.3%)	26.6%	27.5–25.6	32.3	0.249
Negative	21 (44.7%)	77.%	78–76	58.4	
**Parametrial Involvement**					0.059
Positive	23 (48.9%)	51.6%	52.7–50.4	47	
Negative	24 (51.1%)	46%	47–45	38.4	
**Resection Margin Status**					0.052
Positive	17 (36%)	33.5%	34.6–34.3	30.2	
Negative	30 (64%)	62.9%	63.8–61.9	58.7	
**Pelvic Lymph Nodes Metastases**					0.017
Positive	15 (31.9%)	10%	10.8–9.12	20.2	
Negative	32 (68.1%)	69.5%	68.6–70.3	52.9	

**Table 2:** Treatments, complications, and the current status of the 47 patients included in the study.

		Overall Survival			
	Number (%) or Median (Range)	5-Year Survival Rate	95% CI	Mean Survival (Months)	*p-*Value
**Treatment received prior to pelvic exenteration**					
No prior treatment	11 (24%)	79%	77.8–80.1	41.9	0.098
Prior radical histerectomy + adjuvant radiotherapy	2 (4.%)	0%		16.0	0.688
Prior concomitent definitive radiochemotherapy	34 (72%)	41.2%	42.1–40.2	36.2	0.220
**Adjuvant treatment following pelvic exenteration**					
Adjuvant chemotherapy	13 (23%)	70.1%	71.5–68.6	67.8	0.220
**Type of pelvic exenteration (topographic)**					
Total pelvic exenteration	29 (61.7%)	46.7	47.7–45.6	34.8	**0.025**
Anterior pelvic exenteration	17 (36.2%)	50.3	51.5–49.0	51.3	**0.011**
Posterior pelvic exenteration	1 (2.1%)	100%		24	0.128
**The type of pelvic exenterations concerning the levator ani muscle**					
Supralevatorian	28 (59.6%)	52.1%	53.1–51.0	53.3	**0.021**
Infralevatorian	19 (40.4)	43.9%	46.4–42.6	30.7	0.069
**Type of urinary tract reconstruction**					
Bricker ileal incontinent conduit diversion	46 (98%)	52%	51.2–52.9	27.3	0.718
**Type of intestinal tract reconstruction**					
Colostomy	27 (90%)	53%	52.1–54.1	22.9	0.817
Colorectal anastomosis	3 (10%)	100%		29.1	0.209
**Complications**					
Early complications	18 (38.3%)	20%	20.9–19.01	15.1	**0.007**
Late complications	9 (19.1%)	16.7%	18.1–15.2	20.1	0.815
Surgery time—minutes	300–420 (360)				
Loss of blood—mL	400–1800 (1.100)				
Transfused blood volume, mL	0–1.350 (675)				
Days of hospitalization following pelvic exenteration	19 (10–28)				
Mean follow-up time (months)	44.5 (1–88)				
**Status**					
Dead	22 (46.8%)				
Alive	25 (53.2%)	48.7%	49.5–47.9	49.4	
Patients who answered the quality of life questionnaires	18 (28%)				

**Table 3:** The QoL of the 18 patients who answered the EORTC QLQ-C30 and EORTC QLQ-CX24.

Number of Patients = 18	Items ^~^	Mean Score	SD *	Cronbach’s Alpha Coefficient ^#^
**QLQ-C30**				
**Functioning scales α**				
Physical α	1–5	53.4	13.2	0.64
Role α	6, 7	28.5	20.6	0.69
Cognitive α	20, 25	46.4	21.0	0.84
Emotional α	21–24	37.0	23.1	0.93
Social α	26, 27	51.8	20.8	0.90
Global quality of life α	29, 30	32.6	16.1	0.66
**Symptom scales and/or items γ**				
Fatigue γ	10, 12, 18	60.0	16.8	0.58
Nausea and vomiting γ	14, 15	13.2	14.0	0.07
Pain γ	9, 19	73.2	20.0	0.60
Dyspnea γ	8	36.5	22.4	NA
Sleep disturbance γ	11	88.5	22.5	NA
Appetite loss γ	13	43.7	19.7	NA
Constipation γ	16	41.9	19.1	NA
Diarrhea γ	17	13.2	17.9	NA
Financial impact γ	28	90.2	21.9	NA
**QLQ-C24**				
Symptoms Experience γ	31–37, 39, 41–43	21.8	12.1	0.71
Body Image γ	45–47	34.1	28.1	0.88
Sexual/Vaginal Functioning γ	50–53	NA	NA	NA
Lymphoedema γ	38	77.1	27.2	NA
Peripheral Neuropathy γ	40	29.7	31.2	NA
Menopausal Symptoms γ	44	77.7	27.9	NA
Sexual Worry γ	48	72.3	28.8	NA
Sexual Activity α	49	NA	NA	NA
Sexual Enjoyment α	54	NA	NA	NA

* Standard deviation; **^~^** Numbers match the item numbers in the QLQ-C30 and QLQ-CX24.; **^#^** Cronbach’s alpha coefficient is considered to be a measure of validity and reliability [17]; **α** Scores range from 0 to 100, with a higher score indicating a higher level of functioning; **γ** Scores range from 0 to 100, with a higher score indicating a superior level of symptoms.

## Data Availability

We provide our data for the reproducibility of this study in other centers if such is requested.
